# The effect of soursop‐flower‐enriched fried palm olein on some biochemical and hematological parameters of rats

**DOI:** 10.1002/fsn3.3258

**Published:** 2023-02-19

**Authors:** Valerie Demgne Loungaing, Fabrice Tonfack Djikeng, Gires Boungo Teboukeu, Fabrice Herve Njike Ngamga, Hilaire Macaire Womeni

**Affiliations:** ^1^ Research Unit of Biochemistry, Medicinal Plants, Food Sciences, and Nutrition, Department of Biochemistry, Faculty of Science University of Dschang Dschang Cameroon; ^2^ Institute of Agricultural Research for Development, Foumbot Multipurpose Station Foumbot Cameroon; ^3^ Department of Biochemistry and Molecular Biology, Faculty of Science University of Buea Buea Cameroon; ^4^ Department of Biochemistry, Faculty of Science University of Bamenda Bambili Cameroon

**Keywords:** biochemical and hematological parameters, fried olein, oxidation, soursop flowers, Wistar rats

## Abstract

This work set out to, first, assess the role of soursop flower extracts (SFE) in limiting palm olein oxidation during the production of plantain chips, before ascertaining the effect of these soursop‐flower‐enriched fried palm olein on some biochemical and hematological parameters of rats. The extracts were added to 1.5 kg of oil at 1000, 1400, and 1800 ppm, while BHT at 200 ppm served as a positive control (PO+BHT), and the oil without additives was the negative control (PO). The samples were subjected to 15 frying cycles. Total oxidation values varied between 5.94 ± 0.0 and 31.58 ± 0.37; 8.08 ± 0.25 and 28.24 ± 0.00 and 13.71 ± 0.24 and 42.71 ± 0.40 respectively for palm olein enriched with SFE, for PO+BHT and for PO. Twenty‐one groups each comprising five rats received, through dietary supplementation, oils subjected to 0, and 5, 10 and 15 frying cycles for a duration of 30 days. The alanine transaminase and aspartate transaminase of rats fed with oils enriched with SFE at fresh states and at 5 frying cycles was comparable to that of the neutral control group (23.45 ± 2.65 and 93.10 ± 3.53 U/L) and lower than that of the negative control group (52.15 ± 2.01 and 124.07 ± 1.89 U/L). The HDL cholesterol of these animals was also comparable to that of the neutral control group (67.82 ± 4.06 mg/dl) and higher than that of the negative control group (50.25 ± 5.20 mg/dl). White blood cells and mean corpuscular volume of rats fed with fried olein previously enriched with SFE were lower than those fed with fried olein without additives. These extracts are recommended as natural antioxidants for the stabilization of palm olein.

## Graphical abstract

Animals fed with the different fried oils enriched with soursop flower extracts presented a decrease in serum transaminase and creatinine concentration followed by an increase in HDL cholesterol and total serum protein concentrations compared to animals fed with non‐enriched fried oil. An improvement in hematological parameters was also observed through a decrease in white blood cell count and mean corpuscular hemoglobin in rats fed with soursop flower extract enriched fried oil compared to those fed the fried oil without additive.

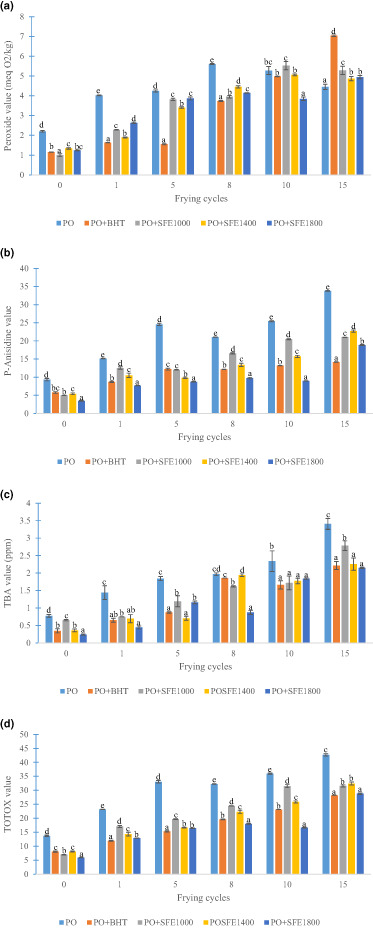

## 1 INTRODUCTION

Vegetable oils are unavoidable food products, as they not only constitute important sources of energy for the body but consist of essential nutrients such as fat‐soluble vitamins (A, D, E, K) and unsaturated fatty acids of the omega 3 (ω‐3 or n‐3), and omega 6 (ω‐6 or n6) families (Ahmad et al., 2021). The omega 6 family (ω‐6 or n6), in particular, are essential for the proper functioning of organisms as they on the one hand, perform a structural role, as building blocks of all cell membranes, and, on the other hand, regulate vital physiological functions such as reproduction, smooth muscle contraction, blood clotting, inflammation, neuronal activity (Leray, 2020). The consumption of adequate proportions of these nutrients is also associated with improving brain and visual functions, and combating cardiovascular diseases (Djuricic & Calder, 2021; Leray, 2020; Mukhametov et al., 2021). Unfortunately, inappropriate culinary treatments such as prolonged frying to which vegetable oils are generally exposed lead to the oxidation of these unsaturated fatty acids, resulting in the formation of oxidation products including hydroxyl radicals, hydroperoxides, alkyls, alkoxyls, aldehydes, alcohols, and ketones (Duguma & Abebaw, 2020; Fadda et al., 2022). All these compounds are very toxic because once ingested through the fried food, they can cause various types of pathologies such as inflammations, arteriosclerosis, cancer, and other diseases related to oxidative stress (Domínguez et al., 2019). In fact, aldehydes such as formaldehyde, acetaldehyde, 4‐hydroxy‐trans‐2‐nonenal and hexenal have neurotoxic and pro‐inflammatory properties (Grootveld, 2022). In addition, animal experiments have shown that the consumption of deep‐fried vegetable oils has hepatotoxic and nephrotoxic effects (Ambreen et al., 2020; Amsalu et al., 2020). The investigations of Syamsunarno et al. (2020) and Islam et al. (2019) also show that the ingestion of thermooxidized dietary oils by rats compromises their health status, resulting in leukocytosis and an increase in serum concentrations of total cholesterol, LDL‐cholesterol, and triglycerides followed by a decrease in HDL‐cholesterol.

The use of antioxidants remains an effective means in the fight against oxidation because they have the ability to donate an electron or a hydrogen atom to free radicals in order to stabilize them, and this leads to an inhibition of the reaction (Al‐Mamary & Moussa, 2021). Antioxidants are naturally present in virgin oils in the form of tocopherols, carotenoids or polyphenols (Fadda et al., 2022), but, unfortunately, they are destroyed during the refining of these oils. In order to overcome this problem, the food industry supplements refined oils with synthetic antioxidants such as butylated hydroxytoluene (BHT), butylated hydroxyanisole (BHA) and therbuthyl hydroquinone (THQ), and propyl gallate (PG) (Fatourehchi, 2020; Lourenço et al., 2019), which unfortunately are very toxic compounds. Indeed, studies have shown that prolonged consumption of BHA and BHT causes hepatotoxicity, DNA damage, gastrointestinal tract damage and tumor cell formation (Fatourehchi, 2020; Lourenço et al., 2019; Xu et al., 2021). These setbacks have, at least, in part, led to growing interest in using natural antioxidants instead of these chemical additives.

So far, several research works (Djikeng, Womeni, Enti et al., 2017; Djikeng et al., 2022; Li et al., 2020; Mahmud & Muhammad, 2021; Şahin et al., 2020) have concluded that natural antioxidants from various plant matrices such as extracts of ginger rhizomes, rosemary leaves, olive leaves, soursop flowers, cinnamon, mangosteen, turmeric, and cassumunar ginger powders have the potential to limit the oxidation of vegetable oils during storage and frying. The findings from these research works also converge on the premise that the stabilizing activity of these natural antioxidants is closely related to the amount and nature of the phenolic compounds they contain. Studies conducted on Soursop Flower Extracts (SFEs) have revealed that this matrix has a total phenolic compound content of 51.33 mg/g GAE (Womeni et al., 2016), while phytochemical analysis detected the presence of vanillic acid, caffeic acid, gallic acid, ferulic acid, and quercetin which are phenolic compounds with good antioxidant activities. Furthermore, Djikeng, Womeni, Enti et al. (2017) showed that these extracts can limit the oxidation of palm olein during storage at 180°C after 24 h of heating.

Based on our search, no work has explored neither the stabilizing effect of soursop flower extracts in palm olein during deep frying, nor the harmful effects of fried oleins on some biochemical and hematological parameters of rats, a task which this paper seeks to accomplish.

## 2 MATERIALS AND METHODS

### 2.1 Materials

Soursop flowers were collected from a multipurpose research station of the Institute of Agricultural Research for Development (IARD) in Foumbot‐Cameroon, in September 2019. Palm olein without additives was purchased from SCS/RAFCA in Bafoussam‐Cameroon, in November 2019.

### 2.2 Methods

#### 2.2.1 Preparation of extracts

The protocol of Djikeng, Womeni, Marrapu et al. (2017) was used with some modifications. The collected soursop flowers were cleaned and dried in an oven at a temperature of 50°C for 48 h. The dried flowers were ground to a texture that would enable it to filter through a 1 mm sieve. The 250 g of powder obtained was macerated in 1 L of methanol at room temperature for 48 h with regular stirring. After that, the extract was filtered with a Wattman No. 1 filter paper. Then, the filtrates were subjected to rotary evaporation at 40°C under reduced pressure, for the removal of solvent, and the extracts obtained were placed in an oven at 45°C for 48 h to reduce traces of the solvent.

#### 2.2.2 Incorporation of palm olein with soursop flower extracts and BHT

The mixture of palm olein with soursop flower extracts and BHT was done according to the protocol of Fabrice et al. (2017) with some modifications. For this purpose, the already well liquefied and homogenized palm olein without additives was divided and introduced into five opaque bottles (1.5 kg of oil per bottle). The crude extracts and BHT were first dissolved individually in 5 ml of methanol with vortexing. Methanolic extract of soursop flowers was added separately into the first three bottles at concentrations of 1000, 1400, and 1800 ppm respectively. In the fourth bottle, BHT (synthetic antioxidant) was introduced at its legal limit concentration of 200 ppm (Metzner et al., 2020) and represented the positive control. The fifth bottle did not receive any additive except 5 ml of methanol. Once the different oil samples were made up, they were shaked regularly for 3 h before being placed uncovered in an oven at 45°C for 24 h for maximum evaporation of the solvent. All oil samples (stabilized and non‐stabilized) were prepared under the same conditions.

#### 2.2.3 Frying of plantain chips

The frying process was done according to the protocol of Leong et al. (2010) with some modifications. One hundred gram of fresh oil was collected first before the frying process. Palm olein sample was heated at 180°C in an electric fryer, and then 50 g of unripe plantain previously cleaned and cut into strips was introduced. After 3 min, the plantain chips were removed from the oil. Then, the hot oil left to cool at room temperature for 5 h before the collection of 100 g of the sample for analysis. The pre‐cooled oil was used to frying another batch of plantains without adding new oil. The operation was repeated 4, 7, 9, and 14 times in order to obtain oil frying at 5, 8, 10, and 15 times. All oil samples (stabilized and non‐stabilized) were fried under the same conditions.

#### 2.2.4 Measurement of oxidation parameters of the oil samples

The measurement of oxidation parameters was done according to standard methods as follow:

Peroxide value: spectrophotometric method of IDF 74 A: 1991 (IDF, 1991).

Anisidine value: official AOCS Cd 18‐90 “p‐anisidine value” method (AOCS, 2003).

Thiobarbituric acid value: method of Draper and Hadley (1990).

TOTOX (total oxidation value) = 2IP + IAn (Shahidi & Wanasundara, 2008).

#### 2.2.5 Treatment of animals

Animals were treated according to the protocol of Zeb and Khan (2019) with some modifications. One hundred five *albino Wistar* rats were individually divided into 21 groups of five rats each. They weighed between 150 and 168 g. They were acclimatized for 1 week according to the rearing conditions prescribed by OECD (2008). The ambient temperature of the animal house was 25°C with a 12‐h light/dark cycle. They had free access to water and food. The staple food (SF) for the animals was composed according to the food composition for animals of Doungue et al. (2020) as follows: maize meal (68%), soybean meal (20%), fish meal (10%), bone meal (1%), cooking salt (0.8%), and vitamin complex (0.1%). The neutral control group received the staple food. The other groups received the corresponding oil sample by dietary supplementation of 2% (2 ml oil in 100 g food). Table 1 shows the distribution of the groups of animals and the composition of their diets.

**TABLE 1:** Food composition of the different animal groups.

Groups	Codes	Diet
1	Neutral control	Staple food (SF)
2	PO	SF + Palm Olein without additives at 0 frying time
3	PO+BHT	SF + Palm Olein enriched with 200 ppm BHT at 0 frying time
4	PO+SFE1000	SF + Palm Olein enriched with 1000 ppm soursop flower extract at 0 frying time
5	PO+SFE1400	SF + Palm Olein enriched with 1400 ppm soursop flower extract at 0 frying time
6	PO+SFE1800	SF + Palm Olein enriched with 1800 ppm soursop flower extract at 0 frying time
7	5PO	SF + Palm Olein without additives at 5 frying time
8	5PO+BHT	SF + Palm Olein enriched with 200 ppm BHT at 5 frying time
9	5PO+SFE1000	SF + Palm Olein enriched with 1000 ppm soursop flower extract at 5 frying time
10	5PO+SFE1400	SF + Palm Olein enriched with 1400 ppm soursop flower extract at 5 frying time
11	5PO+SFE1800	SF + Palm Olein enriched with 1800 ppm soursop flower extract at 5 frying time
12	10PO	SF + Palm Olein without additives at 10 frying time
13	10PO+BHT	SF + Palm Olein enriched with 200 ppm BHT at 10 frying time
14	10PO+SFE1000	SF + Palm Olein enriched with 1000 ppm soursop flower extract at 10 frying time
15	10PO+SFE1400	SF + Palm Olein enriched with 1400 ppm soursop flower extract at 10 frying time
16	10PO+SFE1800	SF + Palm Olein enriched with 1800 ppm soursop flower extract at 10 frying time
17	15PO	SF + Palm Olein without additives at 15 frying time
18	15PO+BHT	SF + Palm Olein enriched with 200 ppm BHT at 15 frying time
19	15PO+SFE1000	SF + Palm Olein enriched 1000 ppm soursop flower extract at 15 frying time
20	15PO+SFE1400	SF + Palm Olein enriched with 1400 ppm soursop flower extract at 15 frying time
21	15PO+SFE1800	SF + Palm Olein enriched with 1800 ppm soursop flower extract at 15 frying time

*Note*: 5, 10, and 15: number of frying time.

Abbreviations: BHT, butylated hydroxytoluene; PO, palm olein; SF, staple food; SFE, soursop flower extract.

The experiment lasted 30 days after which the animals were anesthetized with chloroform vapor. The blood of each animal was collected by heart puncture, and each blood sample was divided into two parts. The first was placed in EDTA tubes for hematological evaluations, and the second was placed in dried tubes and the serum was obtained by centrifugation (1008 *g* for 15 min).

#### 2.2.6 Determination of biochemical parameters

The parameters assessed were transaminases (ALAT/ASAT), total protein, creatinine, triglycerides, total cholesterol, HDL cholesterol, and LDL cholesterol. With the exception of the total protein level which was determined with the BIOLABO kit, all other parameters were obtained from the SPINREACT kits.

#### 2.2.7 Determination of hematological parameters

Hematological analyses were performed on blood samples taken in Ethylene Diamine Tetraacetic Acid (EDTA) tubes by a blood count using an automatic hematological analyzer (SFRI H18 LIGHT auto hematology analyzer).

#### 2.2.8 Statistical analysis

The raw data were analyzed using the SPSS for Microsoft version 26.0. The results were presented as means ± standard deviation. Multiple comparisons were performed using the Waller–Duncan test and the results were considered significant at *p* ˂ .05.

## 3 RESULTS AND DISCUSSION

### 3.1 Chemical characterization of the oils

#### 3.1.1 Peroxide value

**FIGURE 1:**
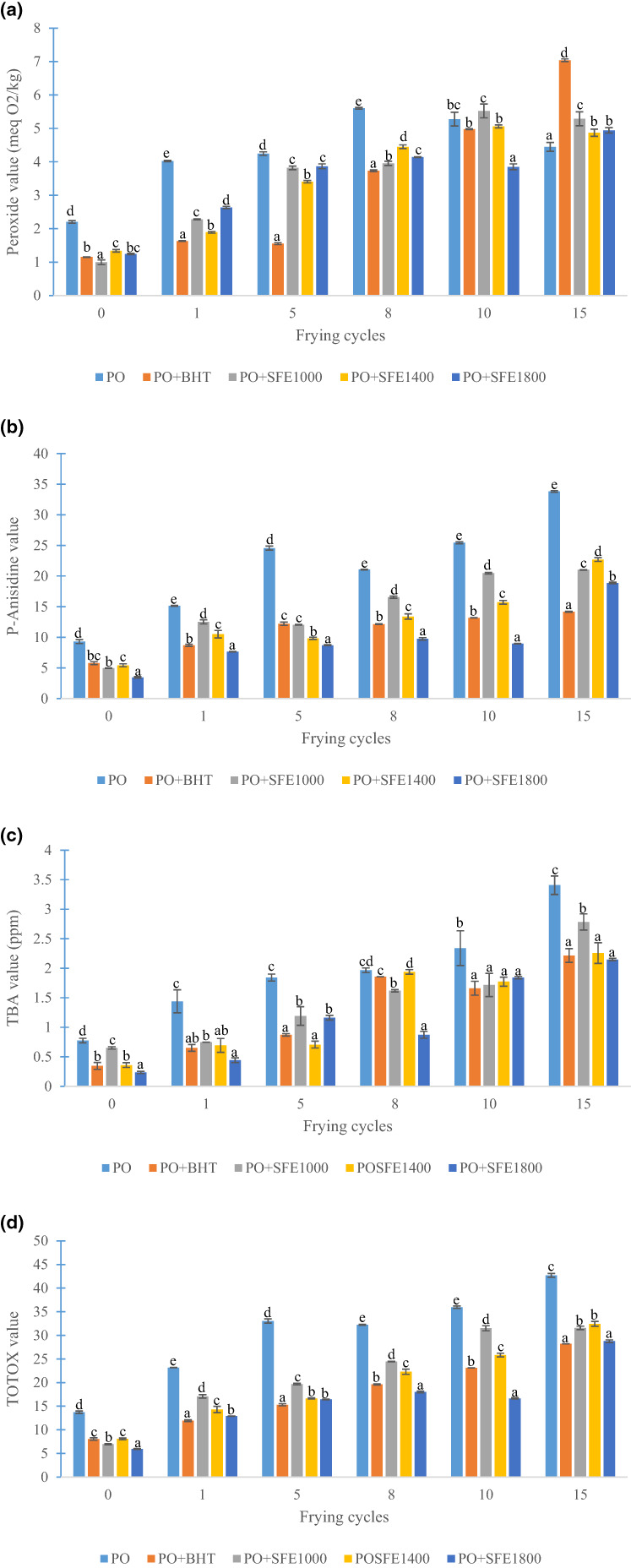
Variation of peroxide value (a), anisidine value (b), thiobarbituric acid value, (c) and total oxidation value (d) of different oil samples during frying. PO: palm olein without antioxidant; PO+BHT: palm olein enriched with butylated hydroxytoluene at 200 ppm; PO+SFE1000: palm olein enriched with soursop flower extract at 1000 ppm; PO+SFE1400: palm olein enriched with soursop flower extract at 1400 ppm; PO+SFE1800: palm olein enriched with soursop flower extract at 1800 ppm. TBA, thiobarbituric acid value; TOTOX, total oxidation value. Data are presented as mean ± standard deviation (*n* = 2). ^a–e^Values of the same frying time with different superscripts differ considerably at *p* < .05.

This parameter provides information on the primary oxidative state of fat. The variation of peroxide value (PV) of all the sample is presented in Figure 1A. In general, the peroxide value of all oil samples increases with the number of frying times, and the sample supplemented with BHT showed the highest peroxide value after 15 frying times (7.3 meq O2/kg). This increase can be linked to the production of hydroperoxides. As has been previously reported, under the effect of heat, unsaturated fatty acids easily lose a hydrogen atom in the α position on their side chains with the formation of alkyl radicals which react with triplet oxygen to produce peroxyl radicals, which will in turn abstract a hydrogen atom from another fatty acid in the medium and then form hydroperoxides (Domínguez et al., 2019; Grosshagauer et al., 2019; Mahmud & Muhammad, 2021). In the current study, the oil samples fortified with soursop flower extracts presented a gradual increase in peroxide value from between the 8th and 15th frying time, and this is attributable both to the good thermal stability and the antioxidant capacity of these extracts. According to Permana et al. (2021), natural antioxidants from plant extracts contain polyphenolic compounds which contribute to their antioxidative activity and can improve the oxidative stability of the oil. However, it should be noted that the peroxide value is not a suitable indicator to determine the level of rancidity of an oil, as high temperatures cause the rapid degradation of hydroperoxides into secondary oxidation products (Djikeng et al., 2022). Fabrice et al. (2017) reported that soursop flower extracts at the concentration of 200–1800 ppm improve the oxidative stability of palm olein during storage at 180°C for 6 days with 4 h of heating per day. In the same light, Asadi and Farahmandfar (2020) found that the *Teucrium polium* extract at the concentration of 200–1000 ppm reduce the formation of hydroperoxides in the canola oil during the frying of potato chips.

#### 3.1.2 *p*‐anisidine value and thiobarbituric acid value

The anisidine value and thiobarbituric acid value help to measure the secondary oxidation products, which are 2, 4‐dienals, 2‐alkenals, and malondialdehydes, respectively. As seen in Figure 1B,C, the anisidine value and thiobarbituric acid value of all oil samples increased significantly (*p* < .05) with the number of frying cycles. Nevertheless, the oils enriched with soursop flower extracts exhibited only a slight increase in these two parameters compared to the negative control (PO). Similarly, the formation of secondary oxidation products in oil samples enriched with soursop flower extracts decreased significantly (*p* ˂ .05) with increasing extract concentration. This means that the activity of these extracts is concentration‐dependent. The increase in the anisidine value and thiobarbituric acid value during frying could be the result of the decomposition of the primary products into secondary ones. Indeed, the termination phase is marked by the breaking of adjacent double bonds of hydroperoxyls followed by the formation of secondary reaction compounds such as hydrocarbons, aldehydes, alcohols, and ketone which are non‐radical stable products (Mahmud & Muhammad, 2021; Viana da Silva et al., 2021). The low increase in secondary oxidation products observed in oil samples enriched with plant extracts might be due to the action of the polyphenolic compounds present. According to Womeni et al. (2016) these soursop flower extracts contain a wide range of phenolic compounds (vanillic acid, caffeic acid, ferulic acid, quercetin, and ellargic acid) with very high antiradical activity and ferric ion reducing power. These compounds would therefore act by giving up their labile hydrogen to the alkyl radicals and peroxides, thus transforming them into more stable non‐radical products. Similar observations by Djikeng et al. (2022) reveal that the protective effect of ginger root extracts in palm olein during accelerated stockage is concentration‐dependent. These results are also in tandem with those of Li et al. (2020) who have reported that rosemary extracts at 2% protect soybean oil by reducing the formation of secondary oxidation compounds during the frying of potatoes chips.

#### 3.1.3 Total oxidation value

Figure 1D shows the variation in TOTOX value of the different oil samples during frying. It can be seen that this parameter increased significantly (*p* < .05) in all samples. However, the oil samples supplemented with extracts showed resistance to rancidity compared with the control without additives (PO). In addition, palm olein enriched with 1800 ppm of extract showed a small increase in TOTOX value compared with the positive control (PO+BHT). The high TOTOX value in the control (PO) can be explained by the absence of antioxidants. This absenceis a consequence of the massive and rapid formation of primary and secondary oxidation products within the frying bath. However, the slight increase in this parameter observed in oils enriched with plant extracts would be linked to the antioxidant action of the flavonoids and phenolic acids present. This is because under frying conditions, phenolic compounds offer good oxidative stability to the oil (Wu et al., 2019). Moreover, the antioxidant power of a compound depends not only on the massive presence of hydroxyl (OH) groups but also on the position of these groups on the aromatic skeleton. When they are in *ortho* or *para* position, the bond dissociation energy of O‐H is low, making it easier to transfer the proton to the free radical in a bid to deactivate it (Al‐Mamary & Moussa, 2021). It follows from the work of Womeni et al. (2016) that the phenolic compounds present in soursop flower extracts have one or more *ortho* or *para* hydroxyl groups. These outcomes are in accordance with those of Ali et al. (2019) who indicated that rice bran oil significantly retards the process of lipid oxidation in soybean oil during frying. Similar finding was done by Okhli et al. (2020), they have reported that citron peel extract enhances stability of sunflower oil during a storage of 5 days at 65°C.

### 3.2 The effect of the consumption of different oil samples on some biochemical parameters of rats

#### 3.2.1 Transaminases

The determination of the serum activity of transaminases (Alanine transaminase and Aspartate transaminase) can offer insights into the pathological state of the liver. These enzymes are found mainly in the cytosol of hepatocytes and are released after hepatocellular damage (Islam et al., 2019). The variations in serum transaminase activities of the different groups of animals are presented in Table 2. It can be seen that the groups of rats fed with the diet supplemented with fresh oil samples enriched with plant extracts showed low serum transaminase concentrations similar to those of the neutral control group. The consumption of the different fried olein samples resulted in a significant (*p* ˂ .05) increase in these parameters in all groups of animals. However, the groups consuming the fried oleins without additives showed the highest levels of serum activities. The increase in serum activity of transaminases in the different groups following the consumption of fried oleins could reflect damages of the liver membrane caused by the oxidative compounds present in these oils. This is because free radicals have the ability to not only cause lipid peroxidation in hepatocellular membranes, but impair its functioning, as well as modify its fluidity and permeability with compromised enzyme activity (Ambreen et al., 2020), resulting in an abnormal release of transaminases into the bloodstream. The low level of serum transaminase activity in the groups fed with oils enriched with plant extracts could be explained on the one hand by the low rancidity of these oil samples, as previously observed with oxidative parameters, and on the other hand, due to the benefits linked to the consumption of phenolic compounds which would have had a hepatoprotective effect. According to Saha et al. (2019), phenols and flavonoids offer protection against oxidative damage to cell membranes by releasing their hydrogen or electrons to free radicals. These results are in agreement with those of Zeb and Khan (2019) who showed that alpha tocopherol minimized the effects of lipid oxidation in the liver of rats after the consumption of oxidized olive oil. The findings are also in line with those of Islam et al. (2019), who found that consumption of fried mustard oil leads to elevated serum transaminase activities.

**TABLE 2:** Effect of different oil samples on ALAT/ASAT, creatinine, and total protein concentration in serum animals

Animal's groups	Parameters			
	ALAT (U/L)	ASAT (U/L)	S‐CREA (μmol/L)	S‐PROT (g/L)
Neutral control	23.45 ± 2.65^a^	93.10 ± 3.53^abc^	69.78 ± 4.65^efgh^	60.35 ± 4.49^fgh^
PO	39.72 ± 1.81^fgh^	99.75 ± 4.94^cd^	65.13 ± 4.65^cde^	53.35 ± 3.03^cde^
PO+BHT	31.67 ± 1.14^cde^	91.17 ± 3.23^ab^	56.76 ± 2.08^ab^	61.95 ± 3.38^fgh^
PO+SEF1000	26.42 ± 0.62^ab^	94.50 ± 2.55^abc^	53.97 ± 2.54^a^	62.69 ± 2.27^fgh^
PO+SEF1400	26.95 ± 0.00^abc^	89.25 ± 4.98^a^	52.10 ± 2.08^a^	65.86 ± 3.23^h^
PO+SEF1800	25.55 ± 0.35^ab^	93.80 ± 5.85^abc^	52.10 ± 3.89^a^	64.52 ± 3.43^h^
5PO	52.15 ± 2.01^j^	124.07 ± 1.89^f^	76.30 ± 2.54^ij^	51.54 ± 4.89^cd^
5PO+BHT	39.90 ± 2.66^fgh^	105.35 ± 2.52^d^	61.41 ± 5.09^ab^	58.48 ± 5.08^efg^
5PO+SEF1000	29.40 ± 0.82^bcd^	93.97 ± 4.97^abc^	66.99 ± 2.54^cdef^	57.04 ± 2.23^def^
5PO+SEF1400	25.72 ± 2.52^ab^	96.25 ± 3.76^bc^	67.92 ± 2.54^def^	62.93 ± 3.12^gh^
5PO+SEF1800	27.30 ± 0.34^abc^	93.80 ± 1.89^abc^	63.27 ± 2.54^cd^	63.77 ± 2.22^gh^
10PO	61.07 ± 2.86^k^	155.57 ± 4.76^i^	80.02 ± 5.09^j^	48.42 ± 2.57^bc^
10PO+BHT	44.80 ± 0.73^hi^	113.75 ± 3.50^e^	65.13 ± 4.65^cde^	57.04 ± 3.53^def^
10PO+SEF1000	33.25 ± 2.83^de^	113.40 ± 4.69^e^	72.58 ± 2.54^fghi^	57.03 ± 6.37^def^
10PO+SEF1400	31.85 ± 3.63^cde^	114.10 ± 6.38^e^	68.85 ± 3.89^defg^	58.02 ± 1.76^efg^
10PO+SEF1800	29.75 ± 0.33^bcd^	99.40 ± 3.53^cd^	65.13 ± 4.65^cde^	58.01 ± 2.27^efg^
15PO	75.07 ± 0.46^l^	167.47 ± 2.28^j^	96.77 ± 2.08^l^	48.01 ± 4.33^bc^
15PO+BHT	49.87 ± 0.14^ij^	146.47 ± 2.80^h^	69.78 ± 3.28^efgh^	51.17 ± 4.12^c^
15PO+SEF1000	43.57 ± 0.43^gh^	132.47 ± 5.34^g^	89.33 ± 2.08^k^	42.75 ± 2.68^ab^
15PO+SEF1400	39.55 ± 1.79^fg^	121.10 ± 5.19^f^	74.44 ± 4.65^ghij^	41.73 ± 2.58^a^
15PO+SEF1800	36.40 ± 2.94^ef^	105.52 ± 5.41^d^	75.37 ± 2.08^hij^	58.19 ± 3.95^efg^

*Note*: Data are expressed as mean ± SD, *n* = 5. Values for a given group in a column followed by a different letter (a–l) as superscript are significantly different according to Waller–Duncan's multiple comparison test (*p* < .05). Neutral control: group fed with staple food; PO: group fed with palm olein without additives at 0 frying time; PO+BHT: group fed with palm olein enriched with 200 ppm BHT at 0 frying time; PO+SFE1000: group fed with palm olein enriched with 1000 ppm soursop flower extract at 0 frying time; PO+SFE1400: group fed with palm olein enriched with 1400 ppm soursop flower extract at 0 frying time; PO+SFE1800: group fed with palm olein enriched with 1800 ppm soursop flower extract at 0 frying time; 5PO: group fed with palm olein without additives at 5 frying time; 5PO+BHT: group fed with palm olein enriched with 200 ppm BHT at 5 frying time; 5PO+SFE1000: group fed with palm olein enriched with 1000 ppm soursop flower extract at 5 frying time; 5PO+SFE1400: group fed with palm olein enriched with 1400 ppm soursop flower extract at 5 frying time; 5PO+SFE1800: group fed with palm olein enriched with 1800 ppm soursop flower extract at 5 frying time; 10PO: group fed with palm olein without additives at 10 frying time; 10PO+BHT: group fed with palm olein enriched with 200 ppm BHT at 10 frying time; 10PO+SFE1000: group fed with palm olein enriched with 1000 ppm soursop flower extract at 10 frying time; 10PO+SFE1400: group fed with palm olein enriched with 1400 ppm soursop flower extract at 10 frying time; 0PO+SFE1800: group fed with palm olein enriched with 1800 ppm soursop flower extract at 10 frying time; 15PO: group fed with palm olein without additives at 15 frying time; 15PO+BHT: group fed with palm olein enriched with 200 ppm BHT at 15 frying time; 15PO+SFE1000: group fed with palm olein enriched with 1000 ppm soursop flower extract at 15 frying time; 15PO+SFE1400: group fed with palm olein enriched with 1400 ppm soursop flower extract at 15 frying time; 15PO+SFE1800: group fed with palm olein enriched with 1800 ppm soursop flower extract at 15 frying time.

Abbreviations: ALT, Alanine transaminase; AST, Aspartate transaminase; S‐CREA, serum creatinine; S‐PROT, serum protein.

#### 3.2.2 Total proteins

Proteins are biochemical macromolecules involved in both structural and biological functions of organisms and are also among the preferred targets of oxidation products. The details presented in Table 2 show that the consumption of the different oil samples enriched with plant extracts in the fresh state, as well as after 5 and 10 frying cycles led to a non‐significant (*p* ˃ .05) variation in total protein concentration in the different test groups compared with the neutral control group. Nevertheless, a significant (*p* ˂ .05) decrease in this parameter is observed in all the groups that received the different oil samples that underwent 15 frying cycles. In addition to this, the groups of rats that consumed the non‐enriched oil samples presented the lowest (*p* ˂ .05) serum protein concentrations compared with all the other groups. The decrease in protein levels in the different groups of animals can be due to the decrease in the digestibility of these molecules following their oxidation by free radicals. Aldehydes, which are among the major degradation products of fat oxidation, can attack the various amino acids (histidine, proline, tryptophan, cysteine, and tyrosine) contained in proteins, causing their oxidation (Hawkins & Davies, 2019; Kehm et al., 2021). This phenomenon generally leads to the formation of protein aggregates, making them unusable by the organism. These protein aggregates are noticeably involved in the development of proteinopathies, such as Alzheimer's disease, Parkinson's disease, and prion disease (Lévy et al., 2019). The high total protein concentrations recorded in the groups fed with samples of oil enriched with plant extracts would be due to the action of natural antioxidants. The interactions between proteins and polyphenols have been found to improve oxidation resistance capacities (Li et al., 2021). Furthermore, the low concentrations observed in the groups fed with oil samples without additive would be related to the high rancidity of these oils, as previously observed in the chemical characterization tests. These results are consistent with those of Ambreen et al. (2020), who found that the consumption of repeatedly heated mixed vegetable oils results in a decrease in serum total protein concentration in rabbits.

#### 3.2.3 Creatinine

The change in serum creatinine concentration is an indicator of the pathological state of the kidneys. The results of this parameter are presented in Table 2. The groups of rats that consumed the fried oleins fortified with plant extracts showed no significant difference (*p* ˃ .05) in this parameter compared to the neutral control group. The highest serum creatinine concentrations were recorded in groups of animals that had received the different samples of non‐enriched fresh and fried oils. This increase could be the result of an inflammation of the kidneys caused by free radicals formed in these oils during frying. An alteration of the kidneys thus limits the phenomena of glomerular filtration, reabsorption, and tubular excretion during the production of urine, with the consequence of renal insufficiency which can be reflected in an increase in serum creatinine (Guerreiro et al., 2022). The low serum creatinine concentration observed in groups of animals that consumed palm olein enriched with soursop flower extracts is relatable to the low oxidative status of these oils This low serum creatinine concentration can effect the phenolic acids and flavonoids contained in the extracts (Womeni et al., 2016). According to Ashkar et al. (2022), caffeic acid possess anti‐inflammatory, antioxidant, and immunomodulatory effects and can inhibits lipid peroxidation in renal tissues. Furthermore, it has been demonstrated that quercetin attenuates oxidative stress, prevents kidney damage, and inhibits renal inflammation in animal models of diabetic nephropathy (Roumeliotis et al., 2019). Comparable results have been reported by Amsalu et al. (2020) and Li et al. (2019).

#### 3.2.4 Lipid profile

**FIGURE 2:**
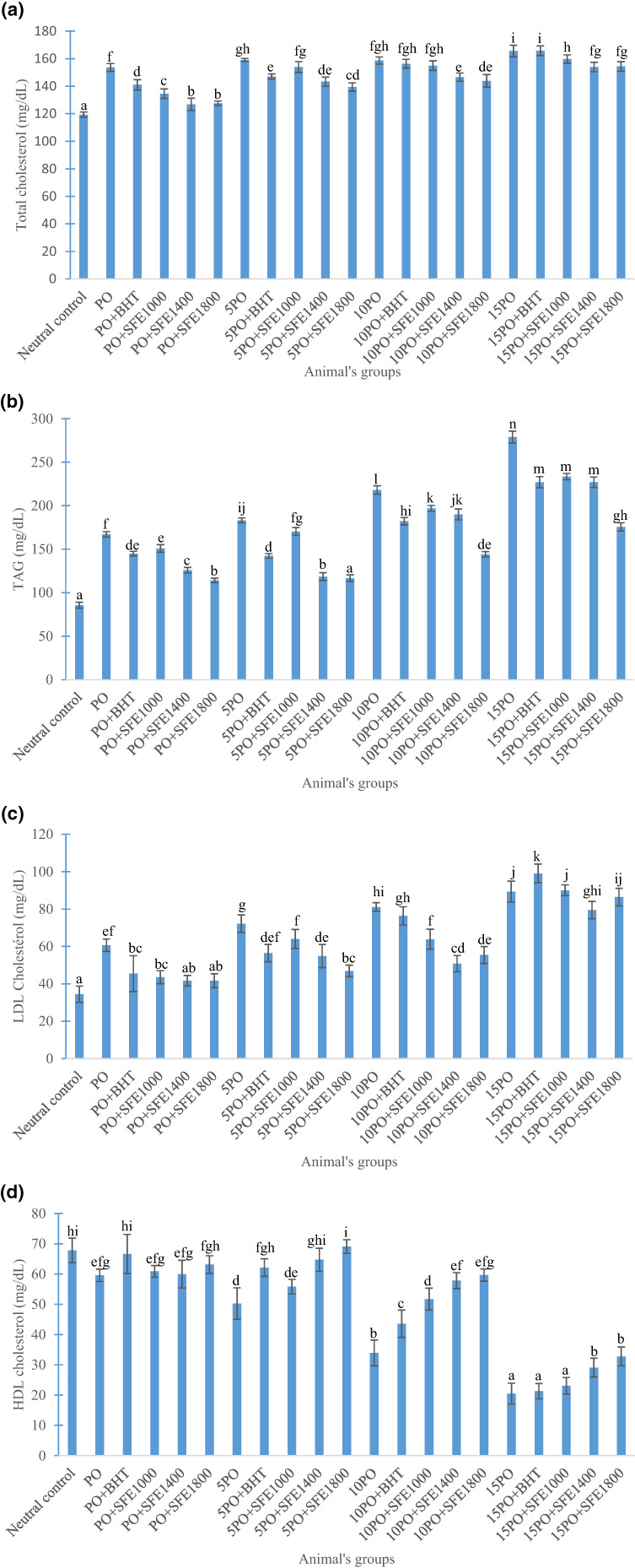
Effect of the different oil samples on the lipid profile of the animals. Total cholesterol (a), triglycerides (b), low density lipoprotein (c), high density lipoprotein (d). Neutral control: group fed with staple food; PO: group fed with palm olein without additives at 0 frying time; PO+BHT: group fed with palm olein enriched with 200 ppm BHT at 0 frying time; PO+SFE1000: group fed with palm olein enriched with 1000 ppm soursop flower extract at 0 frying time; PO+SFE1400: group fed with Palm Olein enriched with 1400 ppm soursop flower extract at 0 frying time; PO+SFE1800: group fed with palm olein enriched with 1800 ppm soursop flower extract at 0 frying time; 5PO: group fed with palm olein without additives at 5 frying time; 5PO+BHT: group fed with palm olein enriched with 200 ppm BHT at 5 frying time; 5PO+SFE1000: group fed with palm olein enriched with 1000 ppm soursop flower extract at 5 frying time; 5PO+SFE1400: group fed with palm olein enriched with 1400 ppm soursop flower extract at 5 frying time; 5PO+SFE1800: group fed with palm olein enriched with 1800 ppm soursop flower extract at 5 frying time; 10PO: group fed with palm olein without additives at 10 frying time; 10PO+BHT: group fed with palm olein enriched with 200 ppm BHT at 10 frying time; 10PO+SFE1000: group fed with palm olein enriched with 1000 ppm soursop flower extract at 10 frying time; 10PO+SFE1400: group fed with palm olein enriched with 1400 ppm soursop flower extract at 10 frying time; 0PO+SFE1800: group fed with palm olein enriched with 1800 ppm soursop flower extract at 10 frying time; 15PO: group fed with palm olein without additives at 15 frying time; 15PO+BHT: group fed with palm olein enriched with 200 ppm BHT at 15 frying time; 15PO+SFE1000: group fed with palm olein enriched with 1000 ppm soursop flower extract at 15 frying time; 15PO+SFE1400: group fed with palm olein enriched with 1400 ppm soursop flower extract at 15 frying time; 15PO+SFE1800: group fed with palm olein enriched with 1800 ppm soursop flower extract at 15 frying time. CHOL‐T, total cholesterol; HDL, high density lipoprotein; LDL, low density lipoprotein; TAG, triglycerides. Data are presented as mean ± standard deviation (*n* = 5). ^a–m^Values of each group differ significantly at *p* < .05.

Changes in lipid profile most often lead to dyslipidemia, which is associated with various diseases such as cancer diabetes and cardiovascular diseases (CVD). Figure 2A–D feature the variations in total cholesterol, triglyceride, LDL‐cholesterol, and HDL‐cholesterol. The experiment showed that the groups of rats that consumed fried palm olein (enriched and non‐enriched) presented a significant increase (*p* ˂ .05) in triglyceride, total cholesterol, and LDL‐cholesterol concentrations followed by a decrease in HDL‐cholesterol compared with the corresponding groups that consumed fresh oils. The groups of animals that received the fried oleins previously enriched with soursop flower extracts showed low concentrations of total cholesterol, LDL‐cholesterol, and triglycerides and an increase in HDL‐cholesterol compared to those that received the fried oils without additives. On the other hand, animals fed with the different oil samples enriched with 1400 and 1800 ppm of plant extracts in the fresh state, as well as after 5 frying cycles showed a non‐significant variation (*p* ˃ .05) in HDL cholesterol compared with the neutral control group. The increase in triglyceride concentration after ingestion of fried oil could be due to the presence of abundant free fatty acids in these oils, and their availability as an esterification substrate in the formation of these molecules. Feleke et al. (2022) and Islam et al. (2019) have also demonstrated that the consumption of fried oleins increase triglyceride levels in rats. The increase in total cholesterol, LDL cholesterol, and the decrease in HDL cholesterol in rats are the consequence of the consumption of oxidation products. In addition, LDL can react with free radicals to form oxidized LDL which when taken up by macrophages accumulate and give rise to foam cells (Jiang et al., 2022; Poznyak et al., 2021). The accumulation of these cells in the interstitial space also contributes to the development of atheromatous plaques followed by the onset of atherosclerosis (Poznyak et al., 2021). Several studies (Ambreen et al., 2020; Feleke et al., 2022; Zeb & Khan, 2019) suggest that the consumption of fried oleins can lead to dyslipidemia. On the other hand, research works have shown that the administration of antioxidants to rats leads to an improvement in their lipid profile. This is because phenolic compounds such as caffeic acid and ferulic acid have the ability to increase plasma HDL cholesterol and reduce LDL and VLDL cholesterol through a reduction of 3‐hydroxy 3‐methylglutaryl coenzyme A (HMGCoA) reductase activity and an increase in plasma lipoprotein lipase and Lecithin Cholesterol Acyl Transfer (LPL and LCAT) activities (Feldman et al., 2021). The 3‐hydroxy 3‐methylglutaryl coenzyme A (HMGCoA) reductase is involved in the synthesis of cholesterol precursors. Both LPL and LCAT play a central role in HDL maturation, and are also involved in determining the composition, and structure (Zeka et al., 2017). Thus, the phenolic compounds present in the extracts could explain the improvement in lipid parameters of animals that consumed the enriched oil samples compared to those that received the non‐enriched oils. These outcomes are in line with those of Zeb and Khan (2019), who demonstrated that alpha‐tocopherol improve the lipid parameters of rats fed with a diet containing thermooxidized sunflower oil.

### 3.3 Effect of oil consumption on the hematological profile of rats

Table 3 shows some hematological parameters of the Wistar rats fed with the different diets. In general, the white blood cells level of the animals varied according to the oxidative state of the consumed oils. The white blood cell concentrations of the animals fed with samples of oils enriched with plant extracts were comparable (*p* ˃ .05) to those of the neutral control group, whereas the animals fed respectively with the non‐enriched oil and the oil containing BHT heated at 15 frying cycles showed significantly high (*p* < .05) white blood cell levels compared to the neutral control group. This indicates that the inflammation is caused by a state of stress in one or more organs by oxidation products. The fact that the consumption of used‐cooking oil resulted in an increase in white blood cell count in animals has also been demonstrated by Syamsunarno et al. (2020). Regardless of the type of oil consumed, no significant difference (*p* ˃ .05) was observed between the red blood cell (RBC), hematocrit (HTC), hemoglobin (HGB) and mean corpuscular hemoglobin concentration (MCHC) levels of all animals in the test and neutral control groups. The results related to mean corpuscular volume (MCV) reveal that all groups of animals fed with the oils containing the plant extracts showed comparable (*p* ˃ .05) MCV to that of the neutral control group. On the other hand, the groups of animals fed with the non‐enriched oils heated to 10 and 15 frying cycles presented the significantly elevated (*p* < .05) MCV compared with the neutral control group. This could reflect macrocytic anemia in these groups of animals. This type of pathology could be the consequence of a vitamin B12 or folate deficiency (Kwon & Park, 2020). It is possible that oxidation products in the oils consumed by these animals caused the inflammations of the distal ileum or jejunum in the small intestine, resulting in poor absorption of vitamin B12, folic acid, and other nutrients. These findings are at variance with those of Zeb and Khan (2019) who discovered that consumption of oxidized olive oil has no significant effect on the mean corpuscular volume of rats.

**TABLE 3:** Effect of different oil samples on the white blood cells, red blood cells, and some figurative elements in the blood of rats

Animal's groups	Parameters					
	WBC (10^3^/μl)	RBC (10^6^/μl)	HGB (g/dl)	HCT (%)	MCV (FL)	MCHC (g/dl)
Neutral control	3.04 ± 1.11^a^	8.96 ± 0.37^a^	16.02 ± 0.76^a^	52.48 ± 1.75^a^	62.86 ± 4.02^abcde^	34.26 ± 3.28^a^
PO	4.26 ± 1.86^ab^	7.50 ± 0.83^a^	15.52 ± 0.59^a^	46.94 ± 2.09^a^	61.92 ± 1.22^abc^	34.18 ± 1.72^a^
PO+BHT	4.26 ± 2.01^ab^	7.29 ± 0.85^a^	15.46 ± 0.87^a^	46.22 ± 4.46^a^	63.08 ± 2.89^abcde^	33.84 ± 2.55^a^
PO+SEF1000	4.18 ± 1.11^ab^	8.00 ± 0.67^a^	16.08 ± 0.39^a^	48.50 ± 3.40^a^	61.92 ± 4.53^abc^	33.94 ± 3.21^a^
PO+SEF1400	4.88 ± 1.96^abcd^	7.46 ± 0.97^a^	15.46 ± 1.17^a^	46.92 ± 2.12^a^	61.46 ± 2.87^abc^	34.76 ± 2.82^a^
PO+SEF1800	3.26 ± 1.70^a^	7.86 ± 0.62^a^	16.00 ± 0.33^a^	48.74 ± 2.20^a^	59.28 ± 1.72^a^	33.52 ± 2.94^a^
5PO	5.24 ± 1.89^abcd^	7.33 ± 1.00^a^	15.40 ± 1.61^a^	45.84 ± 2.03^a^	64.38 ± 2.84^bcde^	34.46 ± 1.31^a^
5PO+BHT	3.90 ± 1.45^a^	7.42 ± 0.96^a^	15.64 ± 1.23^a^	45.42 ± 4.58^a^	60.92 ± 2.48^abc^	33.20 ± 0.89^a^
5PO+SEF1000	4.28 ± 2.30^abc^	7.54 ± 0.72^a^	15.26 ± 1.11^a^	45.98 ± 2.69^a^	59.92 ± 1.79^ab^	33.78 ± 2.01^a^
5PO+SEF1400	5.22 ± 2.99^abcd^	7.77 ± 0.73^a^	16.18 ± 0.31^a^	47.92 ± 2.41^a^	60.08 ± 1.73^ab^	34.00 ± 2.04^a^
5PO+SEF1800	3.86 ± 1.96^a^	7.96 ± 0.61^a^	16.02 ± 0.83^a^	49.16 ± 3.04^a^	60.66 ± 1.78^abc^	33.86 ± 2.33^a^
10PO	7.42 ± 2.69^def^	6.78 ± 0.82^a^	15.80 ± 0.68^a^	47.42 ± 2.04^a^	66.96 ± 5.32^ef^	36.88 ± 2.51^a^
10PO+BHT	7.32 ± 1.40^def^	8.30 ± 0.49^a^	17.72 ± 1.56^a^	53.20 ± 4.65^a^	60.86 ± 2.95^abc^	35.76 ± 2.16^a^
10PO+SEF1000	5.82 ± 0.75^abcde^	8.00 ± 0.50^a^	16.38 ± 0.87^a^	49.88 ± 1.93^a^	60.72 ± 1.53^abc^	34.16 ± 2.47^a^
10PO+SEF1400	5.92 ± 0.76^abcde^	7.46 ± 1.07^a^	16.28 ± 1.54^a^	48.88 ± 4.66^a^	65.18 ± 3.46^cde^	39.64 ± 6.39^a^
10PO+SEF1800	3.80 ± 1.96^a^	7.59 ± 1.02^a^	15.56 ± 1.14^a^	45.12 ± 1.79^a^	66.80 ± 5.32^def^	34.06 ± 1.99^a^
15PO	9.80 ± 2.96^f^	6.02 ± 2.00^a^	13.02 ± 2.84^a^	41.40 ± 3.69^a^	70.44 ± 3.80^f^	39.46 ± 1.75^a^
15PO+BHT	8.24 ± 1.14^ef^	7.74 ± 1.20^a^	15.14 ± 2.47^a^	45.50 ± 7.51^a^	58.34 ± 2.92^a^	36.30 ± 1.68^a^
15PO+SEF1000	8.62 ± 2.53^ef^	6.91 ± 0.56^a^	15.28 ± 0.93^a^	45.34 ± 2.67^a^	62.52 ± 1.77^abcde^	33.74 ± 6.47^a^
15PO+SEF1400	7.26 ± 1.09^cdef^	6.70 ± 0.79^a^	15.20 ± 2.24^a^	43.32 ± 6.20^a^	61.34 ± 2.69^abc^	37.02 ± 0.90^a^
15PO+SEF1800	7.00 ± 1.12^bcdef^	7.84 ± 1.33^a^	16.42 ± 2.64^a^	49.26 ± 4.62^a^	60.30 ± 1.98^abc^	36.60 ± 1.24^a^

*Note*: Data are expressed as mean ± SD, *n* = 5. Values for a given group in a column followed by a different letter (a–f) as superscript are significantly different according to Waller–Duncan's multiple comparison test (*p* < .05). Neutral control: group fed with staple food; PO: group fed with palm olein without additives at 0 frying time; PO+BHT: group fed with palm olein enriched with 200 ppm BHT at 0 frying time; PO+SFE1000: group fed with palm olein enriched with 1000 ppm soursop flower extract at 0 frying time; PO+SFE1400: group fed with Palm Olein enriched with 1400 ppm soursop flower extract at 0 frying time; PO+SFE1800: group fed with palm olein enriched with 1800 ppm soursop flower extract at 0 frying time; 5PO: group fed with palm olein without additives at 5 frying time; 5PO+BHT: group fed with palm olein enriched with 200 ppm BHT at 5 frying time; 5PO+SFE1000: group fed with palm olein enriched with 1000 ppm soursop flower extract at 5 frying time; 5PO+SFE1400: group fed with palm olein enriched with 1400 ppm soursop flower extract at 5 frying time; 5PO+SFE1800: group fed with palm olein enriched with 1800 ppm soursop flower extract at 5 frying time; 10PO: group fed with palm olein without additives at 10 frying time; 10PO+BHT: group fed with palm olein enriched with 200 ppm BHT at 10 frying time; 10PO+SFE1000: group fed with palm olein enriched with 1000 ppm soursop flower extract at 10 frying time; 10PO+SFE1400: group fed with palm olein enriched with 1400 ppm soursop flower extract at 10 frying time; 0PO+SFE1800: group fed with palm olein enriched with 1800 ppm soursop flower extract at 10 frying time; 15PO: group fed with palm olein without additives at 15 frying time; 15PO+BHT: group fed with palm olein enriched with 200 ppm BHT at 15 frying time; 15PO+SFE1000: group fed with palm olein enriched with 1000 ppm soursop flower extract at 15 frying time; 15PO+SFE1400: group fed with palm olein enriched with 1400 ppm soursop flower extract at 15 frying time; 15PO+SFE1800: group fed with palm olein enriched with 1800 ppm soursop flower extract at 15 frying time.

Abbreviations: HCT, hematocrit; HGB, hemoglobin; MCHC, mean corpuscular hemoglobin concentration; MCV, mean corpuscular volume; RBC, red blood cell count; WBC, white blood cell count.

## 4 CONCLUSION

It emerges from this work that soursop flower extracts can enhance the oxidative stability of palm olein during the frying of plantain chips. The effect of these extracts is concentration‐dependent. At 1800 ppm, their efficacy is better than that of BHT. Animals fed with the different fried oils previously enriched with soursop flower extracts showed a decrease in transaminase activities and serum creatinine concentration followed by an increase in HDL cholesterol and total protein compared to animals fed with the non‐enriched fried oils. With regard to hematological parameters, it was evident that the white blood cell count and mean corpuscular volume were significantly lower among rats fed with fried oleins previously enriched with soursop flower extracts, compared to those fed with fried oils without additives. These extracts are thus recommended as natural antioxidants for the stabilization of palm olein.

## CONFLICT OF INTEREST STATEMENT

The authors declare they have not conflict of interest.

## ETHICAL APPROVAL

The animal testing in this work was approved by the Institutional Review Board of the University of Dschang, Cameroon. Ethically, the same body reviewed and approved the protocol and procedures used in this work.

## Data Availability

All data analyzed during this study are included in this published article.
